# An optimized method for *Nasonia* germ-free rearing

**DOI:** 10.1038/s41598-021-04363-9

**Published:** 2022-01-07

**Authors:** Guan-Hong Wang, Robert M. Brucker

**Affiliations:** 1 State Key Laboratory of Integrated Management of Pest Insects and Rodents, Institute of Zoology, Chinese Academy of Sciences, Beijing, 100101 China; 2 Rowland Institute at Harvard University, Cambridge, MA 02142 USA

**Keywords:** Bacteria, Bacterial techniques and applications

## Abstract

A germ-free rearing system is a crucial method for host–microbiota interactions using *Nasonia* as a model system. The previous rearing media in 2012 introduced toxic factors like bleach and antibiotics, required significant effort and volume of media preparation, and the rearing protocols in 2012 and 2016 often resulted in embryos, larvae, and enclosing pupae drowning, underfed, or desiccating. In this work, we optimize the germ-free rearing media that excludes the toxic factors and provide a substrate for the developing animals to have constant access to media without the risk of drowning or desiccation. The new process resulted in an increase in full maturation of larvae to adults from 33 to 65%, with no effect on the rate of growth or final adult size. This significantly improves the applicability of germ-free rearing of *Nasonia* and potentially other parasitoids.

## Introduction

The hymenopteran parasitoid, genus *Nasonia*, is a well-established genetic model for research in evolution, behavior, development, and endosymbiosis^1^. The germ-free rearing (GFR) approach^2,3^ makes *Nasonia* a versatile animal model for host-microbiota interaction^4–6^ as well. However, the previous *Nasonia* Rearing Media (NRM) techniques introduced potential toxic factors like bleach, antibiotics, and/or *Drosophila* medium to the media. What is more, both embryos and pupating larvae are susceptible to drowning at the bottom of the rearing plate, leading to a 35% survival rate from larvae to adults^2,3^. Here we developed an efficient *Nasonia* Rearing Media version 3 (NRMv3) and an optimized germ-free rearing version 2 (GFRv2) protocol.

## Materials and methods

### *Nasonia* rearing and embryo collection

The *N. vitripennis* strain AsymCX(u) (*Wolbachia* free)^7^ was used. All wasps were reared in fly vials with *Sarcophaga bullata* pupae (fly pupae) from Carolina Biological Supply (Burlington, NC) in a 25 °C incubator with constant light. Each fly vial contained 50 female and 15 male wasps with 25 fresh fly pupae. After they were incubated together for 12–24 h, we opened the fly pupae carefully, removing the puparium, and collected the wasp embryos.

### Comparative analysis of development

For the conventional rearing (CV) and NRMv3 with GFRv2 rearing wasp, we collected the 1-day-old female adult and compared their body weight. As the wasp is very small (~ 1.2 mm × 0.4 mm), we observed the mass of the empty 1.5 ml tube and then calculated the difference after putting one female wasp into the tube to calculate the female wasp body weight.

### Survival proportion statistics

We counted the embryo number when we transferred them to the filter (d 1).

We counted the number of dead larvae and pupae remaining on d 20 and calculated the survival rate from larvae to adulthood for each well following the approach from Shropshire et al.^3^:

$$Survival \, rate\,from\,d\,3\,to\,adult= \frac{Number\, of\, d \,3\, larvae-Number\, of\, dead\, larvae\, and\, pupae\, on \,d \,20 }{Number\, of\, d \,3 \,larvae}\times 100$$

## Results and discussion

### *Nasonia* rearing media version 3 (NRMv3)

**Figure 1:**
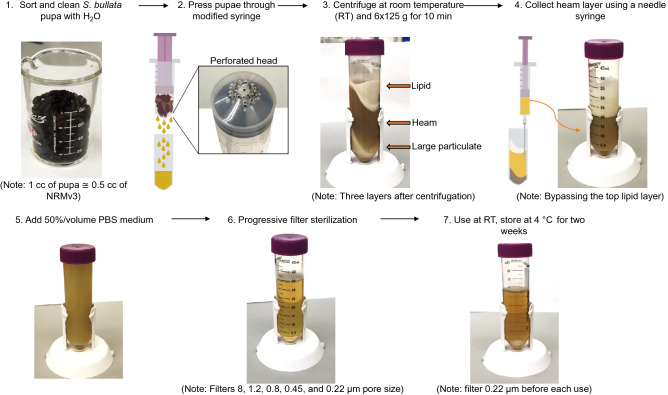
Schematic of the step for *Nasonia* rearing media (NRMv3).

We improved the NRMv3 (Table 1) by modifying a large volume syringe with multiple holes to crush the fly pupae, increasing the fly pupae extract amount by around 10% volume, and decreasing the amount of time spent filtering (Fig. 1). This system works more like an apple press instead of blending and juicing the pupae. We also switched to adding 50% Phosphate Buffered Saline (PBS) by volume instead of Schneider's *Drosophila* medium to the protein extract to exclude the potential unknown effect for wasp development.

**Table 1:** Comparison between NRMv2 and NRMv3, GFRv1 and GFRv2.

*Nasonia* rearing medium version 2 (NRMv2)	*Nasonia* rearing medium version 3 (NRMv3)
1. Fill a sterilized beaker with 150 ml of *S. bullata* pupa. Remove larvae, poor quality pupae, and debris	1. Fill a sterilized beaker with *S. bullata* pupa. Remove larvae, poor quality pupae, and debris
2. In the beaker, cover pupae with sterile millipore water, allow to sit for 1 min, and strain to remove surface particulates from the puparium surface. Some moisture will remain on the pupae	2. In the beaker, cover pupae with sterile millipore water, allow to sit for 1 min, and strain to remove surface particulates from the puparium surface. Some moisture will remain on the pupae
3. Crush the pupae by hand (covered with powder-free nitrile gloves) and squeeze juices through a 100 mm nylon mesh to remove the *S. bullata* puparium	3. Crush the pupae **by 60 ml syringe** with holes bored in the bottom and squeeze extract through a 100 mm polypropylene mesh to remove the *S. bullata* puparium
4. Separate extract (approximately 70–90 ml) evenly into two 50 ml conical tubes and seal tightly	
5. Centrifuge the mixture for 10 min at 4 ℃ (25,000×*g*). The mixture will separate into three distinct layers: a sediment, protein, and lipid layer from bottom to top, respectively	4. Centrifuge the mixture for 10 min at 4 ℃ (25,000×*g*). The mixture will separate into three distinct layers: a sediment, protein, and lipid layer from bottom to top, respectively
6. To prevent clogging during filtration, extract the protein layer using a 22 gauge sterile needle and transfer it to a sterile beaker under sterile laminar flow	5. To prevent clogging during filtration, extract the protein layer using a 22 gauge sterile needle and transfer it to a sterile beaker under sterile laminar flow
7. Add a 2:1 ratio of Schneider's *Drosophila* medium to the protein extract	6. Add a 2:1 ratio of **50% PBS medium** to the protein extract
8. Using a vacuum filtration system, filter the media through progressively smaller pore sizes (11, 6, 2.5, 0.8, and 0.45 um filters) to remove increasingly smaller particulates. To prevent clogging, replace filter paper when flow begins to slow	7. Using a vacuum filtration system, filter the media through progressively smaller pore sizes (8, 1.2, 0.8, and 0.45 um filters) to remove increasingly smaller particulates. To prevent clogging, replace filter paper when flow begins to slow
9. Sterilize the media by filtering through a 0.22 um syringe filter, taking care to use an aseptic technique	8. Sterilize the media by filtering through a 0.22 um syringe filter, taking care to use an aseptic technique
10. Store at 4 ℃ for up to two weeks	9. Store at 4 ℃ for up to two weeks
11. Filter NRM through a 0.22 um syringe filter before use to ensure sterility and remove sedimentation	10. Filter NRM through a 0.22 um syringe filter before use to ensure sterility and remove sedimentation
**Germ-free rearing version 1 (GFRv1)**	**Germ-free rearing version 2 (GFRv2)**
1. *N. vitripennis* strain AsymCx embryos were extracted from *S. bullata* pupae parasitized by virgin females after 12–24 h	1. *N. vitripennis* strain AsymCx embryos were extracted from *S. bullata* pupae parasitized by virgin females after 12–24 h
2. 20–25 embryos were placed on a 3 mm pore transwell polyester membrane (Costar; Corning Incorporated, Corning, NY, USA) and sterilized twice with 70 ml 10% bleach solution and once with 70 ml 70% ethanol solution. The embryos were then rinsed three times with 80 ml sterile millipore water	**2. 20–25 embryos were placed on a circular 100um polypropylene filter, and the filter is rinsed with 200 μl 10% bleach solution once and rinsed with 200 ul 1X PBS once. Then the filter is rinsed with 200 μl 70% ethanol solution once and rinsed with 200 μl 1X PBS three times (d 1)**
3. After rinsing, the transwell insert was moved into a 24 well plate with 250 μ of NRM in the well. All plates were stored in a sterile Tupperware box at 25 ± 2 ℃ in constant light conditions for the duration of the experiment	3. After rinsing, the filter was moved into a 24 well plate with **50 μl ** of NRMv3 in the well. All plates were stored in a sterile Tupperware box at 25 ± 2 ℃ in constant light conditions for the duration of the experiment
4. Under sterile laminar flow, transwells were moved to new wells with 250 ml of fresh NRM every second day	4. Under sterile laminar flow, filters were moved to new wells with **50 ml** of fresh NRMv3 every second day
5. After 11 days, the transwells were moved to dry wells on a clean plate, and the 12 empty surrounding wells were filled with 1 ml of sterile millipore water to increase humidity	**5. After 8 days of fresh NRM3, the filters were kept in the plate and let the larvae develop into pupae**
	**6. After nine to 11 days or check, more than 80% of larvae develop into white or yellow pupae; filters are moved to dry wells in a clean well plate**
	**7. After 13 days, transfer filters to a dry well plate again**

The words in bold are big distinctions between GFRv2 and GFRv1, NRMv3 and NRMv2.

### Optimized germ-free rearing (GFRv2)

**Figure 2:**
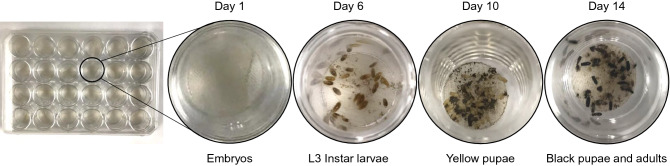
Photos of different stages *Nasonia vitripennis* in the rearing chamber.

We improved the GFRv2 (Table 1) by switching the 3 mm pore transwell polyester membrane $2.55 per each (Thincert for 24-well plate, 8.0 um pore size, translucent, $122.33 for 48/case from USA Scientific, Part Number 5666-2638Q) to 100 um polypropylene filter $0.046 per each (Polypropylene Mesh Sheet, Opaque White, 12″ Width, 24″ Length, 105 microns Mesh Size, 25% Open Area, $25.17 per case which can make 550, 1.4-cm diameter filter from Amazon, Part Number CMP-0105-D) (Fig. 2), which can sharply decrease the cost. In this way, each well only needs 50 μl fresh media instead of 250 μl (GFRv1) each day so we can save 80% of the media. The previous GFRv1 protocol continued feeding the wasps with NRM for 11 days to yellow pupae so that some pupae died in the wet well environment. We continue feeding for only 8 days to avoid introducing NRMv3 when they are in the white pupae stage and are no longer feeding, thus reducing the dead proportion of white pupae.

### Increased survival for larvae to adult

**Figure 3:**
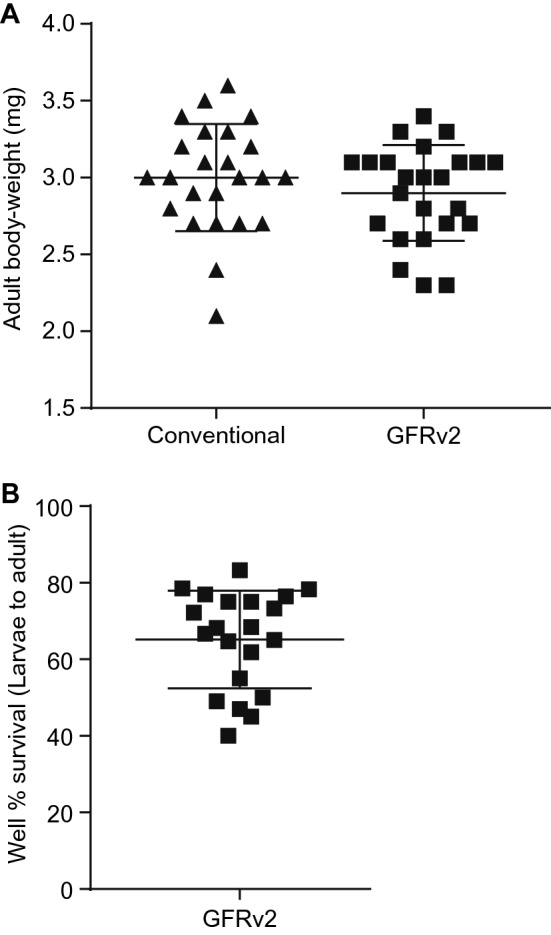
Survival and size of *Nasonia* germ-free adult females. (**A**) One-day-old and germ-free females reared on NRMv3 with GFRv2, and females reared conventionally. Body-weight was not significantly different between germ-free and conventional rearing (Mann–Whitney U test, p > 0.05). Vertical bars with caps represent the standard deviation from the mean. (**B**) The proportion of larval to adult survival in the chamber.

We compared the 1-day-old female body weights between conventional rearing (Wasps were parasitized into *S. bullata* pupae and developed in normal incubator) and optimized NRMv3, and with rearing protocol GFRv2, there are no significant differences (Fig. 3A). The survival rates from larvae to adult using NRMv1^2^ and NRMv2^3^ using GFRv1 were very low—around 25% and 35%, respectively. The optimized NRMv3 with rearing protocol GFRv2 significantly increased the survival rate by approximately 65% (Fig. 3B). Herein, the media NRMv3 with new rearing protocol GFRv2 is a more efficient and cost-effective way for *Nasonia* germ-free rearing.
